# Drainage after Modified Radical Mastectomy – A Methodological Mini-Review

**DOI:** 10.7759/cureus.1454

**Published:** 2017-07-10

**Authors:** George S Stoyanov, Dragostina Tsocheva, Katerina Marinova, Emil Dobrev, Rumen Nenkov

**Affiliations:** 1 Department of General and Clinical Pathology, Forensic Medicine and Deontology, Faculty of Medicine, Medical University – Varna “Prof. Dr. Paraskev Stoyanov”, Varna, Bulgaria; 2 Department of Surgery, Division of Thoracic Surgery, Faculty of Medicine, Medical University – Varna “Prof. Dr. Paraskev Stoyanov”, Varna, Bulgaria

**Keywords:** wound drainage, modified radical mastectomy, breast cancer, methodological review

## Abstract

Breast cancer is a socially relevant group of malignant conditions of the mammary gland, affecting both males and females. Most commonly the surgical approach of choice is a modified radical mastectomy (MRM), due to it allowing for both the removal of the main tumor mass and adjacent glandular tissue, which are suspected of infiltration and multifocality of the process, and a sentinel axillary lymph node removal. Most common post-surgical complications following MRM are the formation of a hematoma, the infection of the surgical wound and the formation of a seroma. These post-surgical complications can, at least in part, be attributed to the drainage of the surgical wound. However, the lack of modern and official guidelines provides an ample scope for innovation, but also leads to a need for a randomized comparison of the results. We compared different approaches to wound drainage after MRM, reviewed based on the armamentarium, number of drains, location, type of drainage system, timing of drain removal and no drainage alternatives. Currently, based on the general results, scientific and comparative discussions, seemingly the most affordable methodology with the best patient outcome, with regards to hospital stay and post-operative complications, is the placement of one medial to lateral (pectoro-axillary) drain with low negative pressure. Ideally, the drain should be removed on the second or third postoperative day or when the amount of drained fluid in the last 24 hours reaches below 50 milliliters.

## Introduction and background

Breast cancer is a socially relevant group of malignant conditions of the mammary gland, affecting both males and females, showing a tendency for development after the third decade and increasing its incidence with age, peaking in the fourth and fifth decade [1-5]. Worldwide it is the most commonly diagnosed malignant condition in females and one of the leading causes for metastatic disease and cancer-related deaths affecting women [1-2, 6-8].

The approach of choice for the treatment of breast cancer is based on its clinical staging. Most commonly the approach of choice is a modified radical mastectomy (MRM), due to it allowing for both the removal of the main tumor mass and adjacent glandular tissue, which are suspected of infiltration and multifocality of the process, and a sentinel axillary lymph node removal [9-10]. This procedure has been shown to have the same long-term effects on survival in breast cancer patients when compared to tissue-sparing techniques in the same stage and grade of the cancer. However, when it comes to local recurrence of the condition MRM has a statistically significant lower incidence of local recurrence of the disease [11-12].

The most common direct post-surgical complications following MRM are the formation of a hematoma, the infection of the surgical wound and the formation of a seroma. These direct post-surgical complications can, at least in part, be attributed to the drainage of the surgical wound [13].

However, the lack of modern and official guidelines and recommendations for drainage of the surgical wound from leading organizations and unions provides a wide opportunity for personalization of the already approved methods and introduction of new techniques and approaches. This provides an ample scope for innovation and customizations, but also leads to a need for a randomized comparison of the results with the aim of promoting the drainage method with the most favorable patient outcomes – optimal drainage, low levels of post-surgical complications, limited physical and mental traumatism. Institution wise, methods must be financially sound and must not interfere with the deontological aspects of medical servicing.

## Review

Approach to wound drainage after MRM was reviewed based on the approach to the armamentarium of the procedure itself, the number of drains used, drain location, type of drainage system, the timing of drain removal and no drainage alternatives.

Surgical armamentarium

The armamentarium, although not connected to the drainage technique per say, has a high impact on the postoperative period and the amount of tissue agitation, predisposing to wound drainage complications. Overall, the consensus is that the best results are achieved using a harmonic scalpel, when compared to standard electrocautery, which lessens the drainage needs. However, electrocautery also achieves a higher percentage of complications associated to postoperative drainage, when compared to those of cold steel MRM [14-20]. These results may be explained by tissue agitation and the physiological effects of electricity when compared to standard physical aggravation and ultrasound [21-22]. Harmonic scalpel was also associated with a lower rate of skin flap necrosis and decreased patient hospital stay [22].

Number of drains used

The running consensus is that the performance of a single drainage system has the same overall effect as compared to the placement of either two or three separate drains [23-24]. Placing a single drain significantly reduces traumatism and patient discomfort, together with the possibility of postoperative complications. This also allows for an earlier hospital discharge without increasing patient discomfort and emotional trauma.

Drain location

There are multiple options for drainage placement following MRM due to the great volume of surgically created free space. However, drainage placement plays a greater role. Placement in the vector of the gravitational gradient gives a greater performance when compared to the placement of either two or three separate drains against it. Therefore, a pectoro-axillary drainage system is superior to placement in other vectors, be it even placement of more than one drain. The choice of either conventional or vacuum drains has not demonstrated a significant role in the location of drainage placement [23].

Type of drainage system used

When classical and vacuum drainage systems are compared, the consensus is that vacuum drainage systems limit trauma and discomfort for the patient, but they may increase the frequency and volume of seromas [9, 25-28]. However, vacuum systems give considerably better results in terms of incidence of postoperative infections and hematoma formation and thereby allow for an earlier hospital discharge of the patient [29].

When compared to one another vacuum drainage systems with low and high negative pressure, the results showed that systems with low negative pressure gave a lower incidence of seroma and infections of the surgical wound, while also limiting hospital stays [9, 29].

Time of drain removal

Opinions that early removal of the drainage systems limits injuries, infections and time of hospital stay, but increased the incidence of seromas seem to be the reigning ones while considering the time of drainage removal [30-33]. However, there is no unanimous opinion on the optimal time for the removal of the drainage system after surgery. Based on the general results, seemingly the best patient outcome with the least complications occurs when the drains are removed on the second or third postoperative day, or preferably when the amount of drained fluid in the last 24 hours reaches below 50 milliliters

Drainage and tissue sealant combination

Some authors recommend a combination of a drainage system and a tissue sealant to further try and decrease the possibility of postoperative complications and hospital stay for patients [34-37]. However, based on the limited report and their discouraging reported figures, it seems that the combination of tissue sealant and a drainage system does not further decrease the duration of hospital stay and the percentage of postoperative complications, mainly seroma [34-37].

No drainage option

Wound closure following the classical and well-known methodologies, without drains has reported a higher frequency of occurrence and greater volumes of clinically recognizable seroma, formed in the postoperative period [38-41]. However, fibrin-based and other type of tissue sealants in the process of closing the surgical wound in some of the procedures have resulted in significantly lower incidence of seroma in those patients compared to other drained and undrained patients [36].

The views that a no drains policy combined with a tissue sealant or quilting flap sutures in MRM, decreases hospital stay, have been expressed by some clinical trials, which have confirmed these encouraging statements in some respects [35-39, 42-46]. The lack of drainage discomfort and pain for the patient, as well as the risk of postoperative infections, have given further commercial rise to these claims. These types of procedures generally allow for an earlier hospital discharge and limit the emotional traumatism for the patient.

Although encouraging, these results are reported only in a small case series, when compared to other options. However, these results seem more encouraging when compared to the drainage and tissue sealant option [32, 35-37]. This approach seems to be the most promising as of the current trends of breast cancer surgery, as it completely excludes the tissue irritation caused by the drainage systems, however, more trials are needed for it to be widely implemented and standardized for this type of procedure.

Compatibility of methods

Despite the accumulated data from various clinical studies, the number and type of drainage systems used after MRM, the drainage approach continues to be determined primarily by the clinical experience and personal preferences of the operating team and the financial capabilities of both the patient and the institution in which the intervention is being carried out. New drainage methods significantly limit the frequency and risk of complications as well as total length of hospital stay, however with significant rise in the total cost due to the high market value of the materials needed and the need for frequent hospital monitoring and outpatient postoperative examinations.

Similar trials and results have also been reported in other fields of surgical medicine, where early removal of drains, when applicable, reduced the hospital stay and postoperative complications in patients [47-50]. However, an individual approach to each patient is highly recommended as there are no standardized drainage protocols, necessitating high surgeon intuition, adaptive to individual cases [46].

While the short-term effects on hospital stay and postoperative complication between all drainage options have been compared, albeit in small cohorts for some methodologies, the long-term effects of drainage on subsequent formation of deep scar tissue and long-term restoration of mobility in these areas have not been compared on a wider scale.

## Conclusions

Currently, based on the general results, scientific and comparative discussions, seemingly the most affordable methodology with the best patient outcome, with regards to hospital stay and postoperative complications, is the placement of one medial to lateral (pectoro-axillary) drain under low negative pressure. The characteristics of the selected method limit postoperative complications such as hematoma formation and wound infection, the discomfort for the patient and period of hospital stay, but however paradoxically may also increase the frequency and volume of clinically recognizable seromas, compared to other methodologies. Ideally, the drain should be removed on the second or third postoperative day or when the amount of drained fluid in the last 24 hours reaches below 50 milliliters. In cases when this drain volume is not achieved, the drain should still be removed prior to the fifth postoperative day.
